# Neurodevelopmental outcomes of very preterm infants born following early foetal growth restriction with absent end-diastolic umbilical flow

**DOI:** 10.1007/s00431-023-05104-y

**Published:** 2023-07-25

**Authors:** Anna Nunzia Della Gatta, Arianna Aceti, Sofia Fiore Spinedi, Silvia Martini, Luigi Corvaglia, Alessandra Sansavini, Mariagrazia Zuccarini, Jacopo Lenzi, Anna Seidenari, Camilla Dionisi, Gianluigi Pilu, Giuliana Simonazzi

**Affiliations:** 1 Department of Medical, Surgical Sciences, University of Bologna, Bologna, Italy; 2 Obstetric Unit, IRCCS AOUBO, Bologna, Italy; 3 Neonatal Intensive Care Unit, IRCCS AOUBO, Bologna, Italy; 4 Department of Psychology “Renzo Canestrari”, University of Bologna, Bologna, Italy; 5 Department of Educational Sciences, University of Bologna, Bologna, Italy; 6 Department of Biomedical and Neuromotor Sciences, University of Bologna, Bologna, Italy

**Keywords:** Foetal growth restriction, Absent end-diastolic flow, Prematurity, Neurodevelopment

## Abstract

This study aims to assess the impact of time of onset and features of early foetal growth restriction (FGR) with absent end-diastolic flow (AEDF) on pregnancy outcomes and on preterm infants’ clinical and neurodevelopmental outcomes up to 2 years corrected age. This is a retrospective, cohort study led at a level IV Obstetric and Neonatal Unit in Bologna, Italy. Pregnant women were eligible if having singleton pregnancies, with no major foetal anomaly detected, and diagnosed with early FGR + AEDF (defined as FGR + AEDF detected before 32 weeks gestation). Early FGR + AEDF was further classified according to time of onset and specific features into very early and persistent (VEP, FGR + AEDF first detected at 20–24 weeks gestation and persistent at the following scans), very early but transient (VET, FGR + AEDF detected at 20–24 weeks gestation and progressively improving at the following scans) and later (LA, FGR + AEDF detected between 25 and 32 weeks gestation). Pregnancy and neonatal outcomes and infant follow-up data were collected and compared among groups. Neurodevelopment was assessed using the revised Griffiths Mental Developmental Scales (GMDS-R) 0–2 years. A regression analysis was performed to identify early predictors of preterm infants’ neurodevelopmental impairment. Fifty-two pregnant women with an antenatal diagnosis of early FGR + AEDF were included in the study (16 VEP, 14 VET, 22 LA). Four intrauterine foetal deaths occurred, all in the VEP group (*p* = 0.010). Compared to LA infants, VEP infants were born with lower gestational age and lower birth weight, had lower arterial cord blood pH and were at higher risk for intraventricular haemorrhage and periventricular leukomalacia (*p* < 0.05 for all comparisons). At 12 months, VEP infants had worse GMDS-R scores, both in the general quotient (mean [SD] 91.8 [12.4] vs 104.6 [8.7] in LA) and in the performance domain (mean [SD] 93.3 [15.4] vs 108.8 [8.8] in LA). This latter difference persisted at 24 months (mean [SD] 68.3 [17.0] vs 92.9 [17.7] in LA). In multivariate analysis, at 12 months corrected age, PVL was found to be an independent predictor of impaired general quotient, while the features and timing of antenatal Doppler alterations predicted worse scores in the performance domain.

*  Conclusion*: Timing of onset and features of early FGR + AEDF might impact differently on neonatal clinical and neurodevelopmental outcomes. Shared awareness of the importance of FGR + AEDF features between obstetricians and neonatologists may offer valuable tools for antenatal counselling and for tailoring pregnancy management and neonatal follow-up in light of specific antenatal and neonatal risk factors.

**Table:** 

**What is Known:** *• Foetal growth restriction (FGR), together with antenatal umbilical Doppler abnormalities, is known to affect maternal and neonatal outcomes.* *• Infants born preterm and growth-restricted face the highest risk for neurodevelopmental impairment, especially when FGR occurs early during pregnancy (early FGR, before 32 weeks gestation).*	
**What is New:** *• The timing of onset and features of FGR and antenatal umbilical Doppler abnormalities impact differently on maternal and neonatal outcomes; when FGR and Doppler abnormalities occur very early, at the limit of neonatal viability, and persist until delivery, infants face the highest risk for neurodevelopmental impairment.* *• Shared knowledge between obstetricians and neonatologists about timing of onset and features of FGR would provide a valuable tool for informed antenatal counselling in high-risk pregnancies.*

**What is Known:**

*• Foetal growth restriction (FGR), together with antenatal umbilical Doppler abnormalities, is known to affect maternal and neonatal outcomes.*

*• Infants born preterm and growth-restricted face the highest risk for neurodevelopmental impairment, especially when FGR occurs early during pregnancy (early FGR, before 32 weeks gestation).*

**What is New:**

*• The timing of onset and features of FGR and antenatal umbilical Doppler abnormalities impact differently on maternal and neonatal outcomes; when FGR and Doppler abnormalities occur very early, at the limit of neonatal viability, and persist until delivery, infants face the highest risk for neurodevelopmental impairment.*

*• Shared knowledge between obstetricians and neonatologists about timing of onset and features of FGR would provide a valuable tool for informed antenatal counselling in high-risk pregnancies.*

## Introduction

Several definitions of foetal growth restriction (FGR) have been proposed over time, but none is really comprehensive of all the different causes leading to FGR [1]. Uteroplacental insufficiency accounts for 25–30% of FGR cases and represents the single most frequent risk factor for FGR [2]. Since its earlier definition, a large number of studies have explored the features and outcomes of FGR, but only few of them have focused specifically on early FGR (FGR occurring before 32 weeks gestation [3]) and examined whether the time of onset and features of early FGR would have a differential impact on pregnancy and neonatal outcomes. It is known that uteroplacental insufficiency with absence of end-diastolic flow (AEDF) in the umbilical arteries detected prior to neonatal viability is associated with a high rate of perinatal deaths and neonatal complications [4]. As there is no feasible in-utero approach to reverse AEDF, the detection of uteroplacental insufficiency at the limit of neonatal viability issues significant challenges for antenatal counselling: in this specific case, antenatal counselling should encompass not only the *pros* and *cons* for maternal health but also the consequences, in the short and long term, of the birth of a preterm and growth-restricted infant. Actually, the effects of FGR expand beyond the neonatal period, as postulated in the “thrifty phenotype” hypothesis [5], and likely include a direct influence on neurodevelopment, leading to cognitive, motor, psychological or behavioural impairment [6–10], as well as an increase in the susceptibility to cardiovascular, metabolic, renal and hepatic diseases [11]. The detrimental effects of FGR on later outcomes are further worsened by prematurity, which is an independent predictor of neurological impairment [10], and by the concomitant evidence of severely impaired flow in the umbilical arteries [12].

At present, it has not been described whether early FGR along with AEDF occurring in the middle of the second trimester, around the limit of neonatal viability, would impact differently on pregnancy and neonatal outcomes compared to early FGR + AEDF occurring few weeks later [1]: thus, the aim of the present study was to specifically assess the impact of the timing of onset and features of early FGR + AEDF on pregnancy outcomes and on preterm infants’ clinical and neurodevelopmental outcomes up to 2 years corrected age (CA).

## Materials and methods

### Ethical approval

The study protocol was approved by the Independent Ethical Committee CE-AVEC, Bologna, Italy (study ID 112/2021/Oss/AOUBo). The study was conducted in conformity with the principles of the Helsinki Declaration. Eligible pregnant women provided written consent to participate to the study for themselves and for their children. Follow-up data for preterm infants were collected within a specific research protocol which had been approved by the same Ethical Committee (study ID EM 193-2018_76/2013/U/Sper/AOUBo).

### Study inclusion criteria: maternal data

A retrospective, cohort study was conducted at a level IV [13, 14] Obstetric and Neonatal Unit in Bologna, Italy. FGR was defined as an absolute foetal size measurement below the 10th centile, in the absence of any congenital anomaly [1]. Pregnant women, having singleton pregnancies, with no major foetal anomaly detected prenatally and estimated foetal weight below 10th centile plus AEDF in the umbilical arteries were included in the study if FGR + AEDF was detected before 32 weeks gestation (early FGR).

As per internal clinical protocol, women diagnosed with early FGR were scheduled for fortnightly antenatal scans, with additional weekly scans in the presence of Doppler abnormalities.

Antenatal evaluations included amniotic fluid volume, foetal biometry and Doppler evaluation of the uterine arteries, umbilical artery, middle cerebral artery and ductus venosus. Amniocentesis was offered to all women diagnosed with FGR, regardless of the gestational week at first detection. When women required hospitalization, a cardiotocographic analysis was also performed. Major criteria for hospitalization were abnormal flow in the ductus venous after 25 weeks gestation, absent amniotic fluid, maternal uncontrolled blood pressure and/or preeclampsia. For each pregnancy, gestational age (GA) was calculated based on the first-trimester crown-rump length.

Ultrasonographic assessment was performed using a Voluson GE Healthcare System machine with a 3.5–5-MHz convex probe. Both umbilical arteries were sampled close to placental insertion, using an insonation angle lower than 30°, including within the sample volume the entire vessel [15].

For the study purpose, early FGR + AEDF was further defined according to GA at first detection: FGR + AEDF detected between 20 and 24 weeks gestation was defined as *very early FGR* + *AEDF* and classified according to persistence of the blood flow anomaly over time into *persistent* (VEP, AEDF first detected between 20 and 24 weeks gestation and persistent in later scans until delivery) and *transient* (VET, AEDF detected between 20 and 24 weeks gestation and progressively improving in the following scans until delivery). FGR + AEDF detected between 25 and 32 weeks gestation (later FGR, LA) served as control group.

### Study inclusion criteria: infant data

Infants were included in the study if born very preterm (GA < 32 weeks) and/or having a birth weight (BW) below 1500 g. As per internal clinical protocol, all preterm infants with these characteristics born at the study centre were admitted to the study Neonatal Intensive Care Unit (NICU) and, following discharge from the NICU, enrolled in a developmental follow-up including periodic assessment of clinical conditions, growth and neurodevelopment up to 24 months CA.

Infants clinical and follow-up data were collected and compared among groups. Since birth, growth was measured using the Intergrowth 21^st^ growth charts [16], which are the most updated standards for measuring postnatal growth in preterm infants from birth to 6 months CA. Since 6 month CA, growth was measured using the World Health Organization (WHO) Child Growth Standards for term newborns, as they overlap with the Intergrowth 21st^st^ charts without the need for any adjustment [17]. Neurodevelopmental assessment was performed at 12 and 24 months CA using the revised Griffiths Mental Developmental Scales (GMDS-R) 0–2 years [18]. The scale evaluates five developmental domains: locomotor (LOC), personal–social (PS), hearing–language (H–L), eye and hand coordination (EH) and performance (PERF), yielding standardized subscale quotients and a general developmental quotient (GQ). GQ was calculated using the tables of standardized scores for the English infants’ population (mean 100.5, standard deviation—SD 11.8), because standardized data for the Italian population are not available. As in previous studies [19] and according to the normative data [18], children’s psychomotor development was defined as normal (GQ score ≥ 88.7), or mildly (GQ score 76.9–88.6), moderately (GQ score 66–76.8) and severely (GQ score ≤ 65) impaired.

#### Statistical analysis

Statistical analyses were performed using IBM SPSS Statistics 28.0 (IBM Corp., Armonk, NY, USA). Data distribution was evaluated through the Kolmogorov–Smirnov test. Since all the follow-up data were distributed normally, parametric tests were used. Differences among the three groups were evaluated using one-way analysis of variance (ANOVA) for continuous data and chi-squared test or Fisher’s exact test—when appropriate—for categorical data. Post hoc comparisons were performed with Student’s *t*-test for continuous variables and Fisher’s exact test for categorical variables, using Bonferroni correction to control the family-wise error rate. Follow-up variables that proved to be significantly different among groups were used to build a set of linear regression models on neurodevelopmental outcomes in which the type of Doppler alteration was included as the independent variable and the neonatal variables which differed among groups as additional covariates. Potential collinearity between independent variables included in the regression models was checked using the Pearson correlation coefficient or the point-biserial correlation coefficient as appropriate. Correlation was defined as “strong” when correlation coefficients were above 0.6. A *p* value < 0.05 was considered statistically significant.

## Results

During the study period (January 2011 to December 2019), all eligible women (pregnant women with FGR + AEDF detected before 32 weeks gestation) were screened. Fifty-five patients fulfilled inclusion criteria: three were excluded from the study as one foetus was also diagnosed with Down syndrome, while two women were lost at follow-up.

Fifty-two pregnant women with a prenatal diagnosis of early FGR + AEDF, diagnosed as previously described, were included in the study (14 in the VET, 16 in the VEP and 22 in the LA group).

Maternal characteristics are reported in Table 1: most pregnant women were Caucasian, 3 were from Africa, and 6 were from Southeast Asia. More than half were primiparae; mean pre-pregnancy BMI was within normal ranges in all groups. No significant difference in maternal variables was detected among groups. As for antenatal scan data (Table 2), in the LA group, both GA and estimated foetal weight at diagnosis were significantly higher compared to both the VEP and the VET group. No significant difference in the examined antenatal scan parameters was detected apart from the ductus venosus α-wave before delivery, which was positive in a significantly larger proportion of infants in the LA group compared to the VEP group. Indications for delivery were significantly different among study groups, with preeclampsia being the most frequent reason for delivery in the LA group, and abnormal umbilical artery flow in the VEP group (Table 2). Amniocentesis was performed upon maternal consent in 27 cases. All obtained karyotypes were euploid.

**Table 1:** Maternal demographic and clinical characteristics within groups. Values are reported as mean [standard deviation] or count (percentage). Differences among groups were non-statistically significant (*p* > 0.05 for all comparisons)

	Transient AEDF 20–24 weeks	Persistent AEDF 20–24 weeks	Later AEDF 25–32 weeks
Pregnant women	14	16	22
*Maternal characteristics*			
Age at diagnosis, years	36.4 [4.6]	35.5 [4.6]	32.4 [6.5]
Caucasian	12 (86%)	11 (69%)	20 (91%)
Black African	1 (7%)	2 (13%)	0 (0%)
South Asian	1 (7%)	3 (19%)	2 (9%)
First pregnancy	7 (50%)	10 (63%)	13 (59%)
BMI during pregnancy, kg/m^2^	27.1 [5.7]	25.8 [4.5]	26.2 [4.9]
Pre-pregnancy BMI, kg/m^2^	24.8 [5.0]	23.7 [3.8]	23.6 [5.0]
Medically assisted reproduction	0 (0%)	1 (6%)	3 (14%)
Blood pressure* Normal values Chronic hypertension Gestational hypertension Preeclampsia	10 (77%) 1 (8%) 0 (0%) 2 (15%)	10 (63%) 4 (25%) 1 (6%) 1 (6%)	8 (36%) 5 (23%) 5 (23%) 4 (18%)
Smoking	1 (7%)	2 (13%)	0 (0%)
Kidney disease	1 (7%)	1 (6%)	2 (9%)
Previous foetal growth restriction	3 (21%)	2 (13%)	4 (18%)
Previous foetal demise	2 (14%)	1 (6%)	1 (5%)
Previous gestational hypertension	3 (21%)	5 (31%)	6 (27%)

*Data available for 51 individuals

*AEDF* absent end-diastolic flow, *BMI* body mass index

**Table 2:** Prenatal scan data at diagnosis and at delivery and indications for delivery. Values are reported as mean [standard deviation] or count (percentage). A *p* value < 0.05 is considered statistically significant

	Transient AEDF 20–24 weeks (VET)	Persistent AEDF 20–24 weeks (VEP)	Later AEDF 25–32 weeks (LA)	*p* value
*Number*	14	16	22	
*Prenatal scan data at diagnosis*				
Gestational age, weeks	23.7 [1.5]	24.0 [1.5]	27.7 [1.5]	** < 0.001**^**a,b**^
Estimated foetal weight, g	438 [203]	432 [180]	787 [209]	** < 0.001**^**a,b**^
Abnormal MCA flow	0 (0%)	3 (19%)	4 (18%)	0.261
RI in the uterine artery	0.77 [0.14]	0.85 [0.32]	0.74 [0.27]	0.440
Notch				
Absent Unilateral Bilateral	1 (7%) 1 (7%) 12 (86%)	2 (13%) 1 (6%) 13 (81%)	3 (14%) 1 (5%) 18 (82%)	1.000
Ductus venosus α-wave				
Positive Negative > 95° centile	11 (79%) 2 (14%) 1 (7%)	13 (81%) 2 (13%) 1 (6%)	18 (82%) 2 (9%) 2 (9%)	1.000
Oligohydramnios	5 (36%)	7 (44%)	5 (23%)	0.379
*Prenatal scan data at delivery*				
Abnormal MCA flow	3 (21%)	7 (44%)	4 (18%)	0.231
Ductus venosus α-wave				
Positive Negative > 95° centile	7 (50%) 7 (50%) 0 (0%)	5 (31%) 5 (31%) 6 (38%)	17 (77%) 4 (18%) 1 (5%)	**0.004**^**a**^
Standstill in foetal growth	2 (14%)	1 (6%)	1 (5%)	0.672
Oligohydramnios	1 (7%)	1 (6%)	1 (5%)	1.000
*Indications for delivery*				
Preeclampsia	2 (14%)	2 (13%)	14 (64%)	**0.001**^**a,b**^
Abnormal umbilical artery flow	8 (57%)	5 (31%)	3 (14%)	**0.022**^**b**^
Abnormal ductus venosus flow	6 (43%)	6 (38%)	1 (5%)	**0.010**^**b**^
Pathologic cardiotocography	5 (36%)	2 (13%)	3 (14%)	0.218

Post hoc comparison (Bonferroni): ^a^VEP vs. LA *p* ≤ 0.05 / 3; ^b^VET vs. LA *p* ≤ 0.05 / 3

*AEDF* absent end-diastolic flow, *MCA* middle cerebral artery, *RI* resistive index

Four intrauterine foetal deaths occurred (three at 27 weeks and one at 29 weeks gestation), all in the VEP group. In all cases, pregnant women were informed about potential benefits and risks of each treatment option and decided not to undergo an iatrogenic delivery, which had been proposed due to the worsening of foetal clinical conditions.

All the 48 infants born to the remaining pregnancies fulfilled the predefined inclusion criteria (GA < 32 weeks and/or BW < 1500 g) and were admitted to the study NICU. None of the infants had any major anomaly detected postnatally. Most infants were born small for gestational age (SGA, birth weight below 10° centile), with no significant differences among groups. Mean (standard deviation [SD]) BW was 789 (285) g and mean (SD) GA was 28.9 (2.1) weeks; infants in the VEP group had significantly lower BW (mean [SD] 636 [191] vs. 884 [273] g, respectively; *p* < 0.05) and GA (mean [SD] 27.6 [1.4] vs. 29.4 [1.9] g; *p* < 0.05) compared to infants in the LA group. Most mothers received a full course of antenatal steroidal prophylaxis (100% in the VET, 83.8% in the VEP and 90.9% in the LA group), with no significant difference among groups (*p* > 0.05). Magnesium sulphate prophylaxis was most likely in the LA group (52.3%, vs. 21.4% in the VET and 25% in the VEP group), but the difference with the other two groups was not significant (*p* > 0.05).

Arterial cord blood pH was significantly lower in infants belonging to the VEP group compared to those in the LA group (mean [SD] 7.16 [0.16] vs. 7.29 [0.09]; *p* < 0.05). A higher incidence of intraventricular haemorrhage (IVH) was detected in the VEP and VET groups compared with the LA group (29% and 25% vs. 0%), although comparisons failed to achieve statistical significance at post hoc evaluation. Similarly, periventricular leukomalacia (PVL) was higher in the VEP group (35%) as compared with both the VET and the LA group (no PVL cases in both groups), but post hoc evaluation did not reach statistical significance, probably due to small numbers. No difference in other clinical morbidities, length of hospital stay or growth parameters at hospital discharge was detected among groups (Table 3).

**Table 3:** Neonatal clinical and growth data. Values are reported as mean [standard deviation] or count (percentage). A *p* value < 0.05 is considered statistically significant

	Transient AEDF 20–24 weeks (VET)	Persistent AEDF 20–24 weeks (VEP)	Later AEDF 25–32 weeks(LA)	*p* value
Pregnant women	14	16	22	
Intrauterine foetal death	0 (0%)	4 (25%)	0 (0%)	**0.010**^**a**^
Infants admitted to the NICU	14	12	22	
Neonatal deaths	5 (36%)	3 (25%)	2 (9%)	0.140
Gestational age (GA), weeks	29.3 [2.4]	27.6 [1.4]	29.4 [1.9]	**0.028**^**a**^
MgSO4 prophylaxis	3/14 (21.4%)	3/12 (25%)	11/21 (52.3%)	0.113
Antenatal steroid prophylaxis	14/14 (100%)	10/12 (83.3%)	20/22 (90.9%)	0.205
Arterial cord blood pH	7.25 [0.08]	7.16 [0.16]	7.29 [0.09]	**0.009**^**a**^
5-min Apgar score	8.1 [1.3]	7.5 [1.4]	8.5 [0.8]	0.066
Birth weight, g	772 [325]	636 [191]	884 [273]	**0.048**^**a**^
Birth weight, SD	− 2.4 [1.8]	− 2.7 [1.5]	− 1.8 [1.2]	0.248
SGA infants	13 (93%)	11 (92%)	15 (68%)	0.153
Birth length, cm	32.4 [4.0]	31.3 (3.4]	34.0 [4.1]	0.163
Birth length, SD	− 2.5 [1.0]	− 1.7 [1.4]	− 1.9 [1.3]	0.237
Birth head circumference (HC), cm	25.3 [2.9]	23.2 [2.3]	25.6 [2.3]	0.062
Birth HC, SD	− 0.9 [1.1]	− 1.5 [1.3]	− 1.0 [1.2]	0.558
Female sex	8 (57%)	5 (42%)	11 (50%)	0.710
Respiratory distress syndrome	5 (36%)	8 (67%)	12 (55%)	0.275
Culture-proven sepsis	0 (0%)	2 (17%)	2 (9%)	0.259
Bronchopulmonary dysplasia	4 (29%)	4 (33%)	3 (14%)	0.374
Patent ductus arteriosus	7 (50%)	6 (50%)	11 (50%)	1.000
Intraventricular haemorrhage	4 (29%)	3 (25%)	0 (0%)	**0.015**^**c**^
Periventricular leukomalacia	0 (0%)	3 (35%)	0 (0%)	**0.013**^**c**^
Retinopathy of prematurity	1 (7%)	1 (8%)	4 (18%)	0.634
Necrotizing enterocolitis	1 (7%)	1 (8%)	0 (0%)	0.288
Length of hospital stay, days	49 [42]	47 [53]	44 [27]	0.921
GA @ discharge, weeks	38.6 [4.0]	39.0 [4.6]	36.8 [2.1]	0.209
Weight @ discharge, g	1836 [304]	1905 [556]	1709 [373]	0.496
Weight @ discharge, SD	− 3.1 [1.6]	− 2.7 [1.1]	− 2.6 [0.7]	0.563
Length @ discharge, cm	41.1 [1.5]	41.1 [4.5]	41.9 [1.8]	0.726
Length @ discharge, SD	− 3.6 [1.1]	− 3.7 [1.1]	− 2.8 [0.8]	0.082
HC @ discharge, cm	32.3 [1.9]	32.8 [2.5]	31.8 [1.4]	0.480
HC @ discharge, SD	− 1.4 [2.2]	− 0.9 [1.3]	− 0.8 [1.0]	0.691

Post hoc comparison (Bonferroni): ^a^VEP vs. LA *p* ≤ 0.05 / 3; ^b^VET vs. LA *p* ≤ 0.05 / 3; ^c^non-significant

*NICU* neonatal intensive care unit, *GA* gestational age, *SGA* small for gestational age (birth weight < 10° centile), *HC* head circumference, *SD* standard deviation

As for follow-up data (Table 4), at 12 months CA, infants in the VEP group had significantly lower weight centile (mean [SD] 3.6 [3.7]) compared to infants in the LA group (mean [SD] 25.6 [21.3]; overall comparison: *p* = 0.018; VEP vs. LA: *p* < 0.05). This difference did not persist at 24 months CA, and no difference in any other growth parameter was detected. Neurodevelopmental assessment through the GMDS-R showed that infants in the VEP group had a significantly lower GQ at 12 months CA compared to those in the LA group (mean [SD] 91.8 [12.4] vs. 104.6 [8.7]; overall comparison: *p* = 0.019; VEP vs. LA: *p* < 0.05); the evaluation of the five developmental domains which build up the GQ revealed that the most compromised domains in the VEP group, compared to the others, were the eye and hand coordination domain (mean [SD]: VEP 93.0 [22.7], VET 91.2 [13.6], LA 109.7 [12.9]; overall comparison: *p* = 0.025, between-group comparison: non-significant) and the performance domain (mean [SD]: VEP 93.3 [15.4], VET 94.3 [13.9], LA 108.8 [8.6]; overall comparison: *p* = 0.008, VEP vs. LA: *p* < 0.05). At 24 months CA, the difference in the performance domain between the two groups persisted (mean [SD]: VEP 68.3 [17.0], VET 78.7 [21.2], LA 92.9 [17.76]; overall comparison: *p* = 0.024, VEP vs. LA: *p* < 0.05), despite no difference in GQ.

**Table 4:** Growth and neurodevelopmental data at 12 and 24 months corrected age. Neurodevelopment was assessed through the revised Griffiths Mental Developmental Scales (GMDS-R) 0–2 years. Values are reported as mean [standard deviation]. A *p* value < 0.05 is considered statistically significant

	Transient AEDF 20–24 weeks (VET)	Persistent AEDF 20–24 weeks (VEP)	Later AEDF 25–32 weeks (LA)	*p* value
Number of infants	14	12	22	
*12 months postmenstrual age*				
Weight, g	7425 [1526]	7471 [602]	8477 [843]	**0.025**^**c**^
Weight, SD	− 2.1 [1.9]	− 2.0 [0.6]	− 0.9 [1.0]	**0.032**^**c**^
Length, cm	67.9 [10.0]	70.5 [1.9]	73.3 [2.3]	0.077
Length, SD	− 2.7 [3.8]	− 1.8 [0.9]	− 0.8 [1.0]	0.114
Head circumference, cm	44.5 [2.4]	44.3 [1.1]	45.1 [1.4]	0.423
Head circumference, SD	− 0.1 [1.6]	− 0.5 [0.8]	− 0.2 [0.9]	0.326
General quotient	92.8 [14.2]	91.8 [12.4]	104.6 [8.7]	**0.019**^**a**^
Locomotor development	82.5 [18.3]	80.3 [19.7]	95.9 [14.4]	0.079
Personal–social development	93.8 [13.0]	94.6 [10.0]	99.5 [12.5]	0.504
Hearing and speech	106.8 [10.7]	101.9 [6.7]	109.9 [12.0]	0.237
Hand and eye coordination	91.2 [13.6]	93.0 [22.7]	109.7 [12.9]	**0.025**^**c**^
Performance tests	94.3 [13.9]	93.3 [15.4]	108.8 [8.6]	**0.008**^**a,b**^
*24 months postmenstrual age*				
Weight, g	10,459 [845]	9846 [1120]	10,724 [913]	0.133
Weight, SD	− 1.2 [0.8]	− 1.7 [0.9]	− 0.9 [0.9]	0.148
Length, cm	84.3 [1.5]	82.4 [3.1]	84.5 [2.5]	0.191
Length, SD	− 0.7 [0.7]	− 1.5 [0.9]	− 0.8 [0.9]	0.174
Head circumference, cm	47.2 [1.7]	46.6 [1.2]	46.9 [1.1]	0.776
Head circumference, SD	− 0.1 [1.2]	− 0.6 [0.9]	− 0.3 [0.9]	0.616
General quotient	93.6 [14.7]	81.1 [16.3]	96.2 [12.0]	0.077
Locomotor development	97.6 [30.3]	86.7 [29.2]	98.3 [24.0]	0.632
Personal–social development	100.9 [14.8]	82.9 [20.1]	97.1 [13.9]	0.090
Hearing and speech	103.0 [10.5]	94.3 [12.2]	98.1 [15.5]	0.498
Hand and eye coordination	90.3 [23.0]	86.7 [13.0]	93.6 [12.8]	0.646
Performance tests	78.7 [21.2]	68.3 [17.0]	92.9 [17.7]	**0.024**^**a**^

Post hoc comparison (Bonferroni): ^a^VEP vs. LA *p* ≤ 0.05 / 3; ^b^VET vs. LA, *p* ≤ 0.05 / 3; ^c^non-significant

*AEDF* absent end-diastolic flow, *SD* standard deviation

Different linear regression models were built up to evaluate the effect of prenatal and neonatal characteristics on each neurodevelopmental outcome which was significantly different among groups (GQ at 12 months CA and performance at both 12 and 24 months CA, Table 5). Collinearity assessment revealed that the only two variables showing a strong correlation were GA and BW (*r* = 0.788, *p* = 0.000); thus, BW was not included, leaving GA, together with arterial cord blood pH, IVH and PVL as the type of Doppler alteration, as covariates to be included into each model.

**Table 5:** Different models evaluating the effect of specific neonatal characteristics on selected neurodevelopmental outcomes: general quotient (GQ) at 12 months corrected age (CA) and performance at both 12 and 24 months CA. A *p* value < 0.05 is considered statistically significant

**Model 1: GQ @ 12 months PMA**					
Parameter	*B*	Standard error	95% confidence interval		*p*
			Min	Max	
*(Constant)*	*76.132*	*196.416*	* − 334.970*	*487.235*	*.703*
Doppler	5.140	2.901	− .933	11.213	.093
Gestational age	− .923	1.761	− 4.609	2.764	.606
Cord blood pH	5.348	29.041	− 55.435	66.131	.856
IVH	4.417	10.792	− 18.171	27.004	.687
PVL	− 20.165	9.112	− 39.238	− 1.093	**.039**

**Model 2: performance @ 12 months PMA**					
Parameter	*B*	Standard error	95% confidence interval		*p*
			Min	Max	
*(Constant)*	*261.808*	*205.587*	* − 168.491*	*692.108*	*.218*
Doppler	8.589	3.037	2.233	14.946	**.011**
Gestational age	.706	1.844	− 3.153	4.564	.706
Cord blood pH	− 27.404	30.397	− 91.025	36.217	.379
IVH	− 12.284	11.296	− 35.926	11.358	.290
PVL	− 7.708	9.538	− 27.671	12.255	.429

**Model 3: performance @ 24 months PMA**					
Parameter	*B*	Standard error	95% confidence interval		*p*
			Min	Max	
*(Constant)*	*112.763*	*353.931*	* − 630.818*	*856.344*	*.754*
Doppler	5.655	4.640	− 4.094	15.404	.239
Gestational age	− 4.154	2.634	− 9.689	1.381	.132
Cord blood pH	11.618	51.479	− 96.535	119.772	.824
IVH	− 29.197	18.280	− 67.601	9.207	.128
PVL	− 24.699	15.143	− 56.514	7.115	.120

*IVH* intraventricular haemorrhage, *PVL* periventricular leukomalacia

As shown in Table 5, having PVL was significantly associated with a lower GQ at 12 months PMA (*p* = 0.039). In addition, Doppler features proved to be an independent predictor of the performance domain at 12 months CA (*p* = 0.011). None of the examined variables proved to be independently associated with the performance domain at 24 months CA in the multivariate analysis.

## Discussion

According to the results of the present study, the timing of onset and course of Doppler alteration, together with relevant neurological morbidities such as PVL, might impact significantly on neonatal outcomes, both during NICU stay and follow-up, in early FGR associated with AEDF. Specifically, infants born to mothers with a diagnosis of early FGR + AEDF detected between 20- and 24 weeks gestation and persistent up to delivery face the highest risk of prematurity-related complications, as they are delivered at lower GAs and with lower BWs compared to infants with early FGR + AEDF detected between 25 and 32 weeks gestation. Furthermore, they are usually born in poorer condition, as documented by the lower arterial cord blood pH, experience more serious neurological complications, including IVH and PVL, in the neonatal period, and have a higher risk for poor growth and impaired neurodevelopment during the first 2 years of life. To note, also infants born to mothers with very early, but transient, AEDF seem to experience a higher risk of neurodevelopmental impairment, even if limited to the performance domain.

Several studies have documented a link between both FGR and SGA status and increased foetal and neonatal mortality and morbidity [20, 21]. In addition, recent systematic reviews and meta-analyses [8–10] have emphasized the high risk of neurodevelopmental impairment in infants with FGR or SGA. In most cases, foetuses with severe FGR are delivered preterm, and this further worsens the risk for adverse outcome. According to the results of the present study, infants with very early and persistent FGR + AEDF face the highest risk of growth and neurodevelopmental impairment and thus should be monitored carefully during both the neonatal period and early childhood. The performance domain, which examines cognitive functions for planning and completing intentional actions and representing objects mentally, appears to be specifically affected, suggesting that close attention should be paid to these functions during infants’ follow-up.

Differences in neurodevelopmental outcomes according to the timing of onset of early FGR + AEDF might be related to a different impact of FGR on the foetus according to the gestational stage in which it occurs; it has been described in animal models that FGR influences brain growth and brain structure, including altered neuronal arborization and reduced number of pre-oligodendrocytes [22]. Furthermore, following FGR, a redistribution of the foetal circulation can occur, in order to maintain an adequate cerebral perfusion (a phenomenon known as “brain sparing”) [23]. Some studies have described a link between brain sparing and adverse perinatal and postnatal outcomes, including neurodevelopment [24, 25], with a direct relationship with the severity of the alteration in the cerebroplacental ratio [26]. Brain sparing is thought to occur regionally rather than globally throughout the brain [23], and this might partly explain the selective impact of FGR associated with AEDF on neurodevelopmental domains. In the present study, no difference in terms of indices of brain sparing was detected among groups, but this result might be linked to the relatively small sample size and deserves further evaluation. Shared information about prenatal indices of brain sparing between obstetricians and neonatologists would add valuable information for antenatal counselling and might improve early neonatal clinical management.

The major point for shared antenatal counselling between obstetricians and neonatologists, when called to counsel couples with early FGR + AEDF detected around the limit of neonatal viability, is that the information provided may trigger a very important decision for the family, leading to different attitudes towards pregnancy continuation or termination. During an antenatal counselling following a diagnosis of very early FGR + AEDF, sharing with the couple evidence-based information about potential maternal and neonatal complications according to timing of onset and features of FGR + AEDF empowers clinicians’ explanation with details that can make the difference in the decision process.

Furthermore, the finding that preeclampsia is the most frequent complication that brings to deliver foetuses in the LA group is important for obstetricians who, in this way, are aware about which patients should be followed carefully with strict laboratory protocols [27].

At present, there is still a lack of potential therapeutic options to prevent neurological impairment following FGR [28]. The knowledge that, among early FGR + AEDF infants, those with FGR + AEDF occurring very early and persisting until delivery face the highest risk for adverse outcomes should prompt further research aimed at discovering biomarkers for identification of uteroplacental insufficiency at its onset; this may deepen our understanding of the complex pathogenetic landscape of FGR, to target potentially reversible or treatable mechanisms of disease (i.e., neuroinflammation). In addition, neonatal research should focus on improving the diagnostic ability of continuous neuromonitoring in the NICU, especially when an infant with early and severe FGR is born extremely preterm.

The strength of the present study relies on the highly selected population, which includes only infants facing the highest risk for severe neonatal and childhood complications (preterm infants who experienced, as foetuses, FGR with AEDF occurring before 32 weeks gestation). The classification according to the timing of onset and features of FGR + AEDF might constitute an additional strength, as it allows to identify these two factors as critical for the following clinical outcome.

As for study limitations, the relatively small sample size and the loss of significance of the AEDF features in the multivariate analysis investigating predictors of GQ at 12 months CA do not allow a thorough generalization of the study results but prompt further research in the same selected population to confirm the actual findings.

## Conclusions

According to the results of the present study, pregnant women with FGR + AEDF detected very early and persisting through pregnancy face the highest risk for intrauterine foetal death. Preterm infants born to these mothers are born at lower GAs and with lower BWs and have higher risk for neurological complications during early life, including IVH and PVL. Furthermore, during the first 2 years of life, these infants might experience worse neurodevelopmental outcomes and poorer growth. The present study provides specific data about growth and neurodevelopment in preterm infants born following early FGR + AEDF during the first 2 years of life, thus offering valuable information for counselling pregnant women who experience this condition.

## Data Availability

The data that support the findings of this study are available on request from the corresponding author, AA.

## Abbreviations

AEDF: Absence of end-diastolic flow
BW: Birth weight
CA: Corrected age
EH: Eye–hand coordination (developmental domain)
FGR: Foetal growth restriction
GA: Gestational age
GMDS-R: Griffiths Mental Developmental Scales
GQ: General developmental quotient
H–L: Hearing–language (developmental domain)
IVH: Intraventricular haemorrhage
LA: Later AEDF + FGR
LOC: Locomotor (developmental domain)
PERF: Performance (developmental domain)
PS: Personal–social (developmental domain)
PVL: Periventricular leukomalacia
SD: Standard deviation
SGA: Small for gestational age (foetus or infant)
VEP: Very early and persistent AEDF + FGR
VET: Very early but transient AEDF + FGR
